# eQTL Viewer: visualizing how sequence variation affects genome-wide transcription

**DOI:** 10.1186/1471-2105-8-7

**Published:** 2007-01-09

**Authors:** Wei Zou, David L Aylor, Zhao-Bang Zeng

**Affiliations:** 1 Bioinformatics Research Center, North Carolina State University, Raleigh, NC 27695-7566, USA; 2 Department of Statistics, North Carolina State University, Raleigh, NC 27695-7566, USA; 3 Department of Genetics, North Carolina State University, Raleigh, NC 27695-7566, USA

## Abstract

**Background:**

Expression Quantitative Trait Locus (eQTL) mapping methods have been used to identify the genetic basis of gene expression variations. To map eQTL, thousands of expression profiles are related with sequence polymorphisms across the genome through their correlated variations. These eQTL distribute in many chromosomal regions, each of which can include many genes. The large number of mapping results produced makes it difficult to consider simultaneously the relationships between multiple genomic regions and multiple expressional profiles. There is a need for informative bioinformatics tools to assist the visualization and interpretation of these mapping results.

**Results:**

We have developed a web-based tool, called eQTL Viewer, to visualize the relationships between the expression trait genes and the candidate genes in the eQTL regions using Scalable Vector Graphics. The plot generated by eQTL Viewer has the capacity to display mapping results with high resolutions at a variety of scales, and superimpose biological annotations onto the mapping results dynamically.

**Conclusion:**

Our tool provides an efficient and intuitive way for biologists to explore transcriptional regulation patterns, and to generate hypotheses on the genetic basis of transcriptional regulations.

## Background

Transcriptional control is a crucial step in organ development and cellular responses to environmental changes. Recent studies have demonstrated that mRNA expression levels vary in both natural and experimental populations [1]. Expression Quantitative Trait Locus (eQTL) mapping seeks to explain such variations by identifying the relationships between the transcript abundance and specific genomic markers [2]. When the transcript abundance is treated as a continuous trait for the purpose of mapping, it is termed an expression trait (eTrait).

eQTL mapping differs from the classical QTL mapping in two important ways, which allow us to ask questions that we cannot address by the traditional approach. First, there is a one-to-one relationship between an eTrait and a gene with its expression profile assayed in the mapping population. The biological information associated with an eTrait gene can be used to suggest potential causal genes for an eQTL. This is important as an eQTL usually spans a large genomic region that contains many genes. For example, eQTL analysis has been used to infer *cis*- and *trans*-acting regulatory regions in yeast [3-5], mice and rat [6-8]. If an eQTL is mapped to a genomic region where the eTrait gene is located, it may suggest the *cis*-regulatory mechanism for the eQTL, *i.e.*, certain sequence variations around the gene region of the eTrait may directly influence the transcript abundance of the gene. In such a case, the genomic region around the eTrait gene can be prioritized for further experimental scrutiny to identify the causal element that affects the eTrait. Otherwise, mapping results may indicate *trans*-acting regulations, *i.e.*, the variation of an eTrait is affected by sequence polymorphisms in other genes. Secondly, the classical QTL mapping focuses on one or a few traits, but an eQTL mapping study may have thousands of eTraits. Biochemical or co-expression relationships among eTrait genes contain additional layers of information beyond just the trait-marker linkages.

To display the myriad of relationships between eTraits, markers, and genes, we need a convenient bioinformatics tool to visualize eQTL mapping results at a variety of scales ranging from a single locus to the entire genome. Additionally, researchers need quick and straightforward ways to integrate these results with the extra information from previous studies on the organism. To address these needs, we have developed eQTL Viewer, a web-based tool that plots eQTL mapping results. The resulting plot displays eQTL for thousands of eTraits in a single view, which makes patterns such as *cis*- and *trans*-regulations readily identifiable. We have also empowered such a plot with the ability to present annotations, highlight features, and organize eTraits in biological groups, such as biochemical pathways. All these characteristics make eQTL Viewer an intuitive and information rich environment to discover and understand genome-wide transcriptional regulation patterns.

## Implementation

eQTL Viewer leverages the power of Scalable Vector Graphics (SVG), an open standard for graphical display recommended by World Wide Web Consortium (W3C) [9]. Instead of creating static graphics, SVG provides a set of instructions to draw dynamic graphics on computer screens. The word 'scalable' is the most important feature of such vector graphics: a large collection of graphic elements can be viewed at a variety of resolutions without sacrificing quality. Using embedded Javascript, virtually unlimited text information can be associated with each graphic element and a user can control the amount of information displayed at one time. These features make SVG well suited for visualizing genomic data, as demonstrated in several bioinformatics applications [10-14].

In eQTL Viewer, each eQTL is represented by a consecutive list of genes that fall within the genomic region where the eQTL is located. An eQTL region can be estimated by a LOD support interval from the mapping procedure [15]. Given a genomic interval, the genes in it can be found using text parsing tools like PERL. We have written example programs to translate eQTL regions into gene lists, and organize them in a XML format file. The eQTL Viewer then converts the XML input into a scalable graph with eTrait genes arranged along the vertical axis and eQTL along the horizontal axis. This basic plot is prevalent in the eQTL literature (see examples in [7]).

Rather than just summarizing the results in a static plot, eQTL Viewer creates an interactive plot that has many useful features. The graph allows a user to zoom in to study genes in each eQTL or zoom out to look at the overall regulation patterns for the genome-wide eTraits. The graph has a search function that can be used to query and highlight an eTrait and its eQTL according to the eTrait gene name. A user can also use customizable scripts to have a list of eTraits and their eQTL highlighted. For example, one can highlight eQTL of all eTraits associated with a certain metabolic pathway. eQTL can also be grouped along the vertical axis according to the biological properties of eTrait genes. This would reveal the eQTL distribution pattern for a number of eTrait genes that share similar biological functions. Each genomic element in the graph can be linked to its annotation information in an external database, such as NCBI. All these features make eQTL Viewer a unique tool for organizing eQTL mapping results and integrating related biological knowledge.

## Results

**Figure 1:**
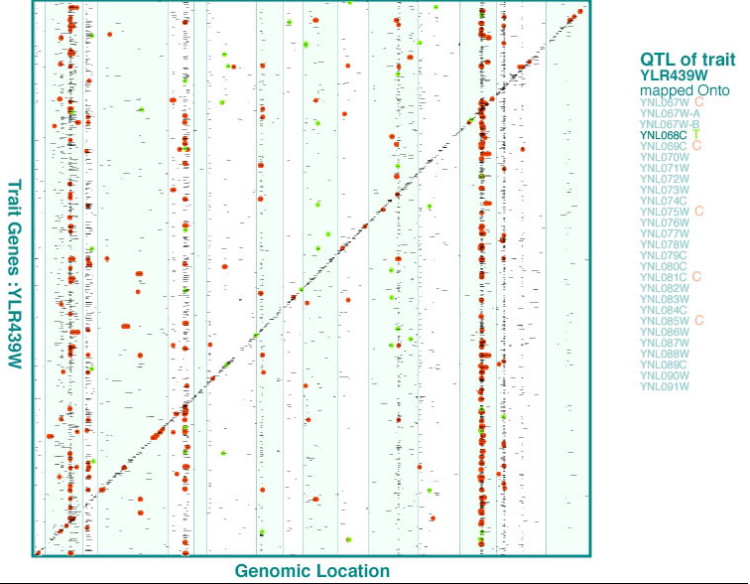
**The display of eQTL information from a yeast study by eQTL Viewer**. A 1.5 LOD support interval around each eQTL peak was considered as an eQTL region. The graph shows all eQTL as small bars. The vertical coordinate of an eQTL corresponds to the genomic location of the eTrait gene, and the horizontal coordinate corresponds to the genomic locations of the candidate genes included in the eQTL region. The interactive graph can be zoomed in to enlarge any particular region of interest. When pointing to a specific eQTL, the names of genes in the eQTL and the name of the eTrait gene appear in the right sidebar and are linked to the yeast genome database. Each green dot indicates that an eQTL contains a Transcriptional factor (the gene followed by a green 'T' in the right sidebar) which has a known binding site at the eTrait gene. Each red dot indicates that a gene (the one followed by a red 'C' in the right sidebar) within an eQTL can form a protein Complex with the eTrait gene product.

As an example, we used our eQTL mapping results obtained from a yeast data set [4] to demonstrate these features. First we applied the multiple interval mapping method [16] to perform eQTL analysis on the data, and found at least one eQTL for 3367, out of the 6195, eTraits. The total number of detected eQTL is 5182. For each eQTL, we used a 1.5 LOD support interval to define the eQTL region. Then we listed genes in each eQTL region and paired them with the corresponding eTrait genes to form 165,285 pairs of eTrait-candidate gene relationships. To visualize these relationships, we generated a scalable and annotated two-dimensional plot (Figure 1) using eQTL Viewer.

The eQTL on the diagonal overlap the genomic regions where the eTrait genes reside, indicating potential *cis*-acting regulations for the eQTL. In Figure 1, there are 736 eTraits with *cis*-acting eQTL along the diagonal (about 12% of the 6195 eTraits assayed). There are also 2969 eTraits with *trans*-acting eQTL off the diagonal (about 48% of all eTraits). There are a number of eQTL "hotspots" formed by *trans*-acting eQTL. Three major ones are located on chromosome 2 (about 450 eTraits), chromosome 14 (about 540 eTraits), and chromosome 15 (about 380 eTraits) (Figure 1). When pointing at an eQTL, the eTrait gene and the candidate genes in the eQTL will be displayed in the right sidebar.

We can superimpose on the graph annotations related with transcriptional regulations. For example, based on the information of yeast transcriptional factors and their binding sites [17], we found 49 eTrait-candidate gene pairs that are parts of the known transcriptional factor regulation network. They are indicated as green dots in Figure 1. Also, we found 734 cases in which a gene within an eQTL region can form a protein complex with the eTrait gene [18]. These are shown in red dots in Figure 1. As there are so many of them, these dots crowd together in the regions where eQTL are dense. In this case, one can zoom in on a cluster to scatter the dots and inspect each eTrait-candidate gene pair as a potential biological mechanism underlying an eQTL. After rearranging eTraits according to KEGG pathways, the graph (available in our website) shows that eTraits of genes in the oxidative phosphorylation pathway [19] have eQTL clustered in the middle of chromosome 15. This suggests a regulatory region for that pathway. Without the grouping feature of eQTL Viewer, such a pattern would not be easily visualized.

A 2-D plot like Figure 1 for mouse eQTL detected by Bystrykh *et al *[6] is also available from our website.

## Discussion

We emphasize gene-gene relationships in eQTL Viewer. These relationships provide important information for biologists to understand and search for the genetic basis of eQTL. An eQTL can span physically a large genomic region, depending on the mapping experimental design. Due to the limitations of linkage studies it is difficult to pin down which gene within an eQTL is the source of eTrait variation [20]. By relating eTraits and genetic markers to their corresponding genes, our eQTL Viewer organizes each eQTL as a list of pairwise relationships between an eTrait gene and the multiple candidate genes in the eQTL region. This goes a step further than just showing the relationships between mRNA probes and polymorphic markers.

Mueller et al. [21] recently introduced their eQTL Explorer package in a similar spirit. While both software packages provide features for exploring eQTL results, they use differing approaches and fulfil complementary functions. eQTL Explorer integrates physiological QTL mapping into eQTL mapping to generate biological hypotheses. Fully recognizing the characteristics of eQTL mapping as compared with classical physiological QTL mapping, we put more emphasis on gene-gene relationships in developing eQTL Viewer as discussed above. Also graphs produced by eQTL Viewer can capture all the eQTL in one plot to display the transcriptional regulatory pattern for the entire transcriptome. Such graphs can be scaled up to hold thousands of eQTL mapped onto a single chromosomal region.

The GenomeGraph module from WebQTL [22,23] is being developed using the same SVG technology to visualize eQTL. It takes the great advantage of WebQTL, an online repository of analysis tools and multiscale mapping data from various model organisms. However, like eQTL Explorer, GenomeGraph visualizes the relationships among probe intensities and genetic markers inferred from eQTL studies. As a standalone visualization tool, our eQTL Viewer allows more flexible incorporation of recent development in statistical methods and biological discoveries. When we interpret an eQTL interval as a list of pairwise relationships between an eTrait gene and multiple candidate genes, additional relationships among genes can be readily plotted on top of the mapping results. Thus, this opens a gate between a single mapping study and the rest of biological investigations in the area.

## Conclusion

eQTL Viewer is a robust web-based bioinformatics tool that generates a scalable graph to visualize estimated relationships between sequence polymorphisms and gene expression profiles. It is our intent to help form a bridge between quantitative genetic analysis and systems biology, and provide a bioinformatics platform to interpret statistical patterns using biological information.

## Availability and requirements

**Project name: **eQTL Viewer

**Project home page: **

**Operating system(s): **Platform independent

**Programming language: **Scalable Vector Graphics, JavaScript

**Other requirements: **To view Scalable Vector Graphics, users may need appropriate plug-in for browsers from Adobe .

**License: **GNU GPL

**Any restrictions to use by non-academics: **Licence needed

## Authors' contributions

WZ conceived the project and SVG output, designed and programmed the web interface, programmed the server-side scripts, co-authored the XML data definition, analyzed the Brem et al [4] data, and co-drafted the manuscript. DLA did programming and testing, co-authored the XML data definition, and co-drafted the manuscript. ZBZ advised on design and features, helped interpret the results from Brem et al [4], and helped draft the manuscript. All authors read and approved the final manuscript.
